# Concomitant Gastric Malignancy and Hepatocellular Carcinoma

**DOI:** 10.7759/cureus.25607

**Published:** 2022-06-02

**Authors:** Reshmi Mathew, Gregory Churchill, Bakht Cheema, Ketav Desai, Ahmad Alkhasawneh, Shiguang Liu, Anwer Siddiqi

**Affiliations:** 1 Internal Medicine, University of Florida College of Medicine – Jacksonville, Jacksonville, USA; 2 Gastroenterology, University of Florida College of Medicine – Jacksonville, Jacksonville, USA; 3 Pathology, University of Florida College of Medicine – Jacksonville, Jacksonville, USA

**Keywords:** stomach ulcer, primary liver lesion, lymphoepithelioma-like carcinoma, epstein- barr virus, hepatitis c virus (hcv), multiple primary malignant neoplasm, hepatocellular carcinoma (hcc), gastric malignancy

## Abstract

Multiple primary malignant tumors (MPMTs) are two or more separate malignancies found at different sites concurrently. Prior studies have shown that the most common tumor associations in MPMTs are typically between two tumors in the digestive system. We present a case of a male patient in his 60s who initially presented with melena and was found to have a clean-based gastric ulcer on initial endoscopic evaluation. Repeat endoscopy on later admission revealed persistent ulceration. Biopsy showed Epstein-Barr virus (EBV) positive lymphoepithelioma-like gastric carcinoma (LELGC), a rare gastric malignancy. The patient underwent endoscopic ultrasound (EUS) for assessment of tumor depth and involvement of perigastric lymph nodes, but was incidentally found to have a liver lesion. Biopsy of the liver lesion was positive for hepatocellular carcinoma (HCC) with no morphologic similarity to the gastric malignancy. This case highlights a rare finding of MPMTs. In addition to the diagnosis of a rare gastric malignancy, the patient developed a well-known but uncommon phenomenon of non-cirrhotic HCC associated with hepatitis C virus (HCV). Due to an increasing number of advances in cancer therapy that are leading to increased survival times, clinicians can expect for a patient to develop MPMTs in their lifetime. A high index of suspicion must exist for the possibility of MPMTs because treatment options and outcomes can be vastly affected by their findings.

## Introduction

Multiple primary malignant tumors (MPMTs) are defined as two distinct primary cancers diagnosed synchronously (within six months) or metachronously (beyond six months) [1]. MPMTs have an incident rate of about 0.52%-11.7% [1]. Even more unique is the diagnosis of two distinct malignancies which are either rare or have developed within a nonclassical pathway. MPMTs occur more commonly in men and older patients (>50 years old) [1]. Furthermore, adenocarcinoma has been the most common pathology identified in synchronous and metachronous cancer groups [1]. Although most MPMTs occur due to random chance [2], patients with genetic susceptibility and those who are survivors of prior cancer confer an increased risk of developing MPMTs [3]. This case highlights the importance of continued follow-up and surveillance in cancer survivor patients, not only to monitor recurrence of disease or metastasis, but also to monitor for the development of a new primary cancer. This case report will expand upon a patient synchronously diagnosed with lymphoepithelioma-like gastric carcinoma (LELGC) and hepatocellular carcinoma (HCC) in the setting of only mild hepatic fibrosis.

## Case presentation

A male patient in his 60s with a history of recent hepatitis C virus (HCV) eradication, severe alcohol use disorder, tobacco use, mild hepatic fibrosis, coronary artery disease, and peripheral arterial disease presented to the ED with epigastric pain and melena concerning for upper gastrointestinal bleed. Initial labs revealed an acute drop in hemoglobin (hemoglobin 5.8 g/dL, hematocrit of 19.2%), severe iron deficiency (ferritin 11.5 ng/mL, serum iron of 12 µg/dL, iron saturation of 3%, total iron-binding capacity (TIBC) 472 mcg/dL, transferrin of 372 mg/dL), and a normal hepatic function panel without coagulopathy. The patient was appropriately resuscitated with fluids and packed red blood cells and underwent esophagogastroduodenoscopy (EGD) which revealed a 3-cm non-obstructing, nonbleeding, cratered gastric ulcer with a clean base. The patient was discharged on a twice-daily proton pump inhibitor (PPI) and scheduled for surveillance endoscopy in eight weeks.

**Figure 1:**
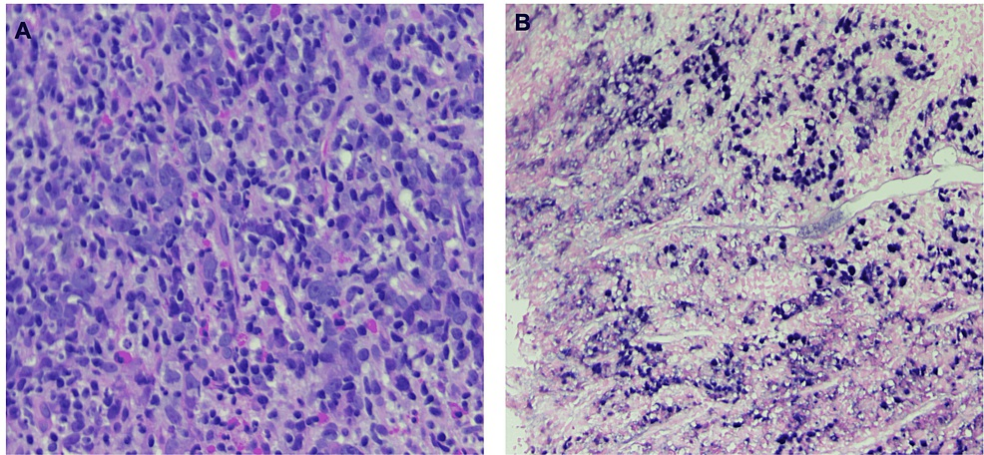
(A) Poorly differentiated large and oval tumor cells with vesicular to clear nuclei, prominent nucleoli and abundant eosinophilic cytoplasm with poorly defined cell borders in dense lymphoid infiltrate in a non-desmoplastic stroma reminiscent of lymphoid tissue (H&E 20x). (B) Poorly differentiated epithelial tumor cells are positive for EBV-encoded RNA (ISH 10x). EBV: Epstein-Barr virus

Despite compliance with PPI therapy, repeat EGD revealed a cratered ulcer with both raised and depressed margins, friable mucosa, and oozing blood with contact suspicious for malignancy. The ulceration was found on the lesser curvature of the stomach, 2 cm from the gastroesophageal junction. The lesion measured 30 mm by 20 mm in maximal cross-sectional diameter. Gastric biopsies revealed poorly differentiated malignant cells reactive for pan-cytokeratin and Epstein-Barr virus (EBV)-encoded ribonucleic acid (RNA) consistent with LELGC (Figure 1A, 1B).

**Figure 2:**
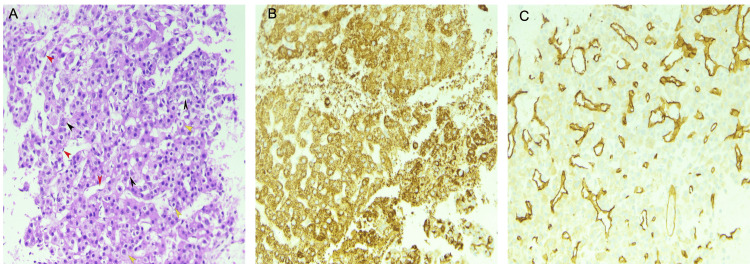
(A) FNA cell block section shows well to moderately differentiated HCC arranged in cords of neoplastic cells, few cells with large pleomorphic nuclei and nucleoli (black arrow) interspersed with clear cells (yellow arrow) and flat endothelial cells wrapping the trabeculae (red arrow) (H&E 10x). (B) Hep Par 1 immunostain positive in HCC tumor cells (IHC 10x). (C) CD34 immunostain highlighting the endothelial cell proliferation in HCC (IHC 10x). FNA: Fine-needle aspiration; HCC: Hepatocellular carcinoma.

**Figure 3:**
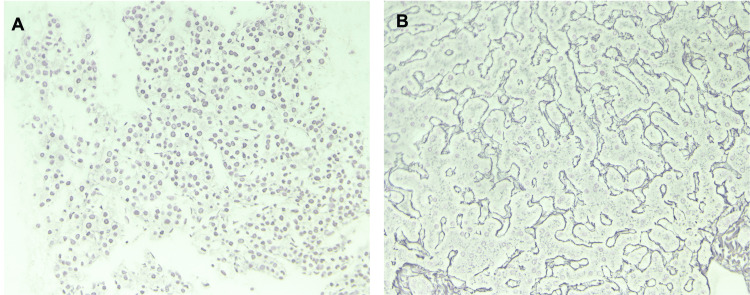
(A) Reticulin stain showing reticulin strands are attenuated and outline large masses of HCC neoplastic cells. (B) In comparison, reticulin-stained benign liver shows the reticulin strands are sharply outlining the normal two-cell thick hepatic cords. HCC: Hepatocellular carcinoma

The patient underwent endoscopic ultrasound (EUS) for assessment of tumor depth and involvement of adjacent lymph nodes, which incidentally revealed a 16 mm by 13 mm hypoechoic lesion in the left lobe of the liver. Fine needle aspiration (FNA) of the liver lesion revealed positive staining for HepPar 1 and CD34 (Figure 2A-2C). Reticulin staining was also performed and showed reticulin highlighting the thickened trabeculae (Figure 3A), consistent with the diagnosis of HCC. Figure 3B shows reticulin staining of normal liver parenchyma for comparison. The liver lesion showed thickened hepatic plates with no morphologic similarity to the gastric lesion.

The patient was deemed a poor surgical candidate due to significant comorbid conditions. It was recommended to follow with oncology for initiation of chemotherapy. However, due to complications related to severe coronary artery disease and peripheral artery disease, the patient was lost to follow-up.

## Discussion

The criteria to diagnose MPMTs were proposed by Warren and Gates, stating that each tumor must have evidence of malignancy on histologic examination, each tumor must be geographically separate and distinct, and the possibility of a metastatic lesion to have spread from a prior cancer must be excluded [1]. The interval period between the diagnosis of a first malignancy and a second malignancy in MPMTs ranged from 1-14 years [3]. Older patients (>50 years of age) and men are found to have the greatest risk of developing MPMTs [1]. One study found that the most frequent location of MPMTs was in the digestive system [1]. Additionally, the most common tumor association in MPMTs was between two tumors in the digestive system [1]. The most common sites of involvement were in the large intestine, stomach, and liver [3], consistent with our patient who had two distinct malignancies in the stomach and liver. It is unclear why MPMTs were more commonly found in the digestive system. It is possible that prior studies of MPMTs occurred in regions where there were environmental and genetic factors that portend a higher chance of developing gastric malignancy. It is also possible that a primary malignancy in the digestive system leads to changes in the microenvironment that make it suitable for the development of a second malignancy.

LELGCs may develop in the stomach, esophagus, lungs, skin, salivary glands, tonsils, thymus, or cervix [4]. When occurring in the stomach, they comprise 1.1-4.6% of all gastric cancers, having an overall more favorable prognosis than other types of gastric cancers [5]. LELGCs can be characterized by two distinct histologic subtypes. The first histologic subtype is characterized by well-defined epithelial nests separated by broad areas of lymphocytic reaction [5]. The second pattern is characterized by tumor cells growing in a diffuse manner that mimics malignant lymphoma [5], similar to the subtype found in our patient. The exact reason for why LELGCs portend a more favorable prognosis compared to other gastric malignancies remains unclear. However, one hypothesis states that lymphocytic infiltration in LELGC is a host defense mechanism against cancer, with a greater degree of lymphocytic infiltration being associated with a better prognosis [6].

LELGCs can be further characterized into two subsets that include those that are EBV-positive versus microsatellite instability (MSI)-high carcinoma [5]. The majority of LELGC cases are caused by EBV, with >80% of cases being EBV-positive [5]. The prevalence of MSI-high carcinoma in LELGC ranges from 7-39% [5]. EBV-positive carcinomas are more commonly found in the cardia and middle portion of the stomach [7], consistent with our patient who had EBV-positive LELGC in the cardia of the stomach. MSI-high carcinomas are more common in the gastric antrum [7]. The diagnosis of LELGC should primarily rely on the detection of EBV by EBV-encoded RNA polymerase chain reaction (PCR) or by the detection of MSI-high status by immunohistochemistry for deoxyribonucleic acid (DNA) repair proteins and/or microsatellite PCR of specific markers [5].

The patient, in this case, had a history of HCV and was status post-treatment with glecaprevir/pibrentasvir. However, one year after treatment, the patient was incidentally found to have a liver lesion consistent with HCC. Classically, HCC occurs in the setting of cirrhosis, but up to 20% of cases occur in non-cirrhotics, most often associated with chronic viral hepatitis (B/C), nonalcoholic fatty liver disease, hemochromatosis, alpha-1 antitrypsin deficiency, aflatoxin exposure, or other toxin exposure [8]. The incidence of HCC in non-cirrhotic patients with HCV ranges from 4.4%-10.6% [9]. Studies have shown that HCC risk is still increased after HCV eradication [8], implying a direct oncogenic effect of the virus. HCV viral proteins have been shown to inhibit tumor suppressor genes and cell cycle checkpoints, causing the increased cell growth and division seen in cancer [10]. Furthermore, significant alcohol use in the presence of HCV synergistically increases the risk of HCC. Despite mild hepatic fibrosis on liver elastography (F0-F1) and HCV eradication in our patient, the cumulative effects of long-standing HCV, significant alcohol use, and tobacco use put him at increased risk for developing HCC. Due to the ongoing risk of carcinogenesis despite the eradication of HCV, there should be active monitoring of nonviral risk factors in patients with prior HCV infection.

When MPMTs are diagnosed, each tumor should be independently evaluated and staged for treatment [11]. Generally, the more aggressive tumor that poses the greatest risk to a patient’s survival and quality of life should be addressed first. If surgery is appropriate for a patient, surgery should be a priority for both tumors and can be combined with chemoradiation or other treatment modalities [11]. The treatment of MPMTs after surgery should be according to the National Comprehensive Cancer Network guidelines for each tumor’s pathologic stage [11].

When considering MPMTs, one must attempt to identify a causal factor linking the carcinomas. We first consider environmental factors such as EBV, HCV, alcohol use, and cigarette smoke in addition to any published genetic syndromes (perhaps involving microsatellite instability) as the causation in our patient. Both primary carcinomas in our patient had their own distinct risk factors present. Based on these findings, there appears to be no obvious connection between the two carcinomas.

## Conclusions

In a time when there are an increasing number of advances in cancer therapy leading to increased survival rates, clinicians can expect to encounter more cases of MPMTs in a patient. Owing to differences in their presentation, rarity, and sometimes difficulty in their diagnosis, MPMTs are often missed or misdiagnosed. This can pose adverse effects on a patient’s overall prognosis if appropriate therapy is not offered. Therefore, this case serves to bring awareness of the possibility of multiple primary cancers in a patient’s lifetime. In this case, the patient was diagnosed with LELGC, a rare gastric malignancy that is often difficult to diagnose. This is due in part to LELGCs often being mistaken for submucosal tumors or lymphoma. The patient was also diagnosed with HCC without a history of cirrhosis, another rare occurrence. Although this patient had multiple risk factors that put him at increased risk for individual malignancies, there appears to be no obvious connection between the two carcinomas based on our findings. To our knowledge, this is the first known case of concomitant LELGC and HCC.
